# Left ventricular geometric patterns in newly presenting nigerian hypertensives: An echocardiographic study

**DOI:** 10.1186/1471-2261-6-4

**Published:** 2006-01-20

**Authors:** Akinyemi Aje, Adewole A Adebiyi, Olulola O Oladapo, Adekola Dada, Okechukwu S Ogah, Dike B Ojji, Ayodele O Falase

**Affiliations:** 1 Department of Medicine, University College Hospital, Ibadan, Nigeria; 2 Department of Medicine, College of Medicine, University of Ibadan, Ibadan, Nigeria

## Abstract

**Background:**

Hypertension is a global problem and it is prevalent in Nigeria. Left ventricular hypertrophy is a major complication of hypertension with risk of sudden death and arrhythmias among others. Abnormal left ventricular geometric patterns also increase the burden of morbidity and mortality. It is therefore important to know the different left ventricular geometric patterns in Nigerian hypertensives because of their prognostic significance.

**Methods:**

One hundred (100) newly presenting hypertensives (53 males and 47 females) and 100 controls (53 males and 47 females) were recruited for the study. All were subjected to clinical evaluation and full echocardiographic examination was performed according to the ASE recommendation. The relative wall thickness and the presence or absence of echocardiographic left ventricular hypertrophy were used to determine the various geometric patterns

**Results:**

The mean age of the hypertensive subjects was 56.06 (± 7.68) years while that of the control subjects was 56.10 (± 7.68) years. There was no significant difference in the mean ages of the two groups. In the hypertensive subjects 28% had normal geometry, 26% had concentric remodeling, 28% had concentric hypertrophy and 18% had eccentric hypertrophy. In the control group, 86% had normal geometry, 11% had concentric remodeling, 3% had eccentric hypertrophy and none had concentric hypertrophy. There was statistical significance when the geometric patterns of the hypertensive and controls were compared (χ^2 ^= 74.30, p value < 0.0001).

**Conclusion:**

The study showed that only 28% of the hypertensive subjects had normal LV geometric pattern while 86% of the normal subjects had normal geometry. There is need for longitudinal studies in order to prognosticate the various geometric patterns.

## Background

Hypertension is a leading non-communicable disease in Nigeria. It has for long been globally recognised as the most prevalent cardiovascular disease. Increased left ventricular (LV) mass is a recognised complication of systemic hypertension and has been shown to be an important predictor of cardiovascular morbidity and mortality in hypertensives[1,2]. Several studies [3-6] have shown the significance of left ventricular geometry in hypertensives. The prognostic importance of different geometric patterns in hypertension had also been established[4,7,8].

Studies had documented significant racial differences in the hypertensive heart[9,10]. However most of the studies had been on Africans in diaspora [11-13] where environmental, social and dietary influence may contribute to the morphological adaptation of their left ventricle. Limited data had emanated from indigenous African population. We therefore sought to determine left ventricular geometric patterns in a native African populace.

## Methods

### Study population

One hundred (100) consecutive, newly presenting adult hypertensive subjects aged 18 and above with blood pressure greater or equal to 140/90 mmHg seen at the cardiac clinic of University College Hospital, Ibadan were enrolled over 6 month period. Normal controls were recruited from among hospital staff, and relatives of hospital patients. Ethical approval was obtained from the institutional ethical review board and informed consent was obtained from the study participants. Subjects with diabetes mellitus, chronic renal failure, valvular heart disease and previous myocardial infarction were excluded from the study.

Blood pressure was measured with a mercury sphygmomanometer (Accosson) according to standard guidelines[14]. Systolic and diastolic blood pressures were measured at Korotkoff sounds phases I and V respectively. Blood pressure was taken on three separate occasions during different hospital visits and the average of the three readings was used for the study.

The study was powered at 90% to detect a 17% difference in the prevalence of LVH between hypertensive subjects and controls at a minimum sample size of 89 subjects.

### Echocardiography

Echocardiographic examinations were done with the patient in partial left lateral decubitus position using an Aloka SSD 1700 machine (Aloka Co. Ltd., Tokyo, Japan) with 3.5 MHz transducer. Two-dimensional guided M-mode measurements were obtained as recommended by the American Society of Echocardiography [15]. Two experienced physicians performed the echocardiography. In our laboratory, the intraobserver concordance correlation coefficient ranged from 0.76 to 0.98 while that of the interobserver concordance ranged from 0.82 to 0.96. Measurements were averaged over 3 cardiac cycles.

Left ventricular mass (LVM) was calculated using the formula[16] that has been shown to yield values closely related (r = 0.90) to necropsy LV weight (Devereux-modified ASE Cube formula).

LV mass (g) = 0.8 (1.04 (IVSd + LVIDd + PWTd) 3 - LVIDd3) + 0.6

Left ventricular mass was indexed by the allometric power of height[17]. Left ventricular hypertrophy (LVH) was considered present if the left ventricular mass index ≥ 46 g/m^2.7 ^(i.e. 2 standard deviations above the mean value for LVMHt^2.7 ^in the control group). Relative wall thickness (RWT) was calculated as 2 × PWTd/LVIDd. Increased relative wall thickness was present when RWT ≥ 0.45 [18]. LV geometry was defined using RWT and LV mass index (LVMI).

Normal geometry – Normal LVMI and RWT

Concentric remodeling-Normal LVMI and Increased RWT

Eccentric LVH – Increased LVMI and RWT < 0.45

Concentric LVH – Increased LVMI and RWT ≥ 0.45

LV ejection fraction (EF) was calculated using Teichholz's formula[19].

### Data analysis

Statistical analysis was performed with SPSS software version 10.0 (SPSS Inc., Chicago, Illinois). Data was expressed as the mean ± SD for continuous variables while frequencies are expressed as percentages. Categorical variables were compared with chi-square while means were compared with "t" test for independent samples. Differences among more than two groups were tested by one way analysis of variance (ANOVA) followed by the Scheffe's post hoc test. Significant P value was taken to be <0.05.

## Results

One hundred hypertensive subjects (53 males and 47 females) and 100 controls (53 males and 47 females) aged 35 to 68 years participated in the study. The clinical characteristics of the subjects are as shown in Table 1. There was no significant difference in the ages of the groups while the body mass index (BMI), heart rate, weight, height, systolic, diastolic, pulse and mean arterial pressures of the hypertensive subjects were significantly more than in the control group.

**Table 1:** Demographic Characteristics of hypertensive subjects and controls

***Variable***	***Hypertensives (n = 100)***	***Controls (n = 100)***	***P value***
Age (years)	56.06 (7.68)	56.10 (7.68)	0.971
Sex (Male/Female)	53/47	53/47	
Heart Rate (bpm)	82.32 (13.43)	80.69 (12.64)	0.38
Weight (Kg)	71.74 (12.32)	67.27 (8.57)	0.003*
Height (m)	1.63 (0.09)	1.66 (0.08)	0.005*
BMI (Kg/m2)	27.29 (4.87)	24.45 (2.70)	<0.0001*
BSA (m2)	1.76 (0.16)	1.75 (0.14)	0.382
SBP (mmHg)	164.54 (15.27)	118.22 (8.10)	<0.0001*
DBP (mmHg)	101.74 (7.13)	70.30 (6.70)	<0.0001*
Pulse Pressure (mmHg)	62.95 (14.57)	47.93 (8.80)	<0.001*

Bpm = beat per minute, BMI = Body mass index, BSA = Body surface area, SBP = Systolic blood pressure, DBP = Diastolic blood pressure, MAP = Mean arterial pressure

* = Statistical significance

Table 2 shows the echocardiographic parameters in both groups. The hypertensive subjects had significantly thicker septal and posterior wall and a larger relative wall thickness. The LVM and the indexation for height^2.7 ^were also significantly more in the hypertensives. The LV geometric patterns of the hypertensive subjects were significantly different from that of the controls.

**Table 2:** Echocardiographic Characteristics in hypertensive subjects and controls

Variable	Hypertensives (n = 100)	Controls (n = 100)	P value
IVSD (cm)	1.08 (0.23)	0.83 (0.12)	<0.0001*
PWTD (cm)	1.05 (0.18)	0.83 (0.11)	<0.0001*
EDD (cm)	4.51 (0.60)	4.53 (0.52)	0.822
ESD (cm)	3.02 (0.56)	2.94 (0.45)	0.295
FS (%)	34.43 (6.18)	35.49 (6.62)	0.246
EF (%)	69.42 (8.74)	72.16 (8.12)	0.02*
Aorta (cm)	2.81 (0.36)	2.85 (0.34)	0.504
AVO (cm)	1.93 (0.27)	2.00 (0.32)	0.09
LA (cm)	3.44 (0.43)	3.06 (0.38)	<0.0001*
RWT	0.48 (0.14)	0.37 (0.07)	<0.0001*
LVM (g)	170.60 (46.05)	121.70 (28.63)	<0.0001*
LVM/HT^2.7 ^(g/m^2.7^)	46.43 (13.35)	31.13 (7.43)	<0.0001*
LV Geometry			<0.0001*
Normal	28	86	
Concentric remodeling	26	11	
Concentric hypertrophy	28	0	
Eccentric hypertrophy	18	3	

IVSD = Interventricular septal thickness in diastole, PWTD = Posterior wall thickness in diastole, EDD = End diastolic diameter, FS = Fractional shortening, EF = Ejection fraction, AVO = Aortic valve opening, LA = Left atrium, RWT = Relative wall thickness, LVM = Left ventricular mass

* = Statistical significance

Age did not correlate significantly with the relative wall thicknesses(r = 0.30, p-value = 0.671) and the left ventricular mass indexed for height^2.7 ^(r = -0.004, p-value = 0.951). Table 3 depicts demographic characteristics of different geometric patterns (both controls and hypertensives). There were significant differences in systolic, diastolic, pulse and mean arterial pressures in all the different geometric patterns. The systolic, diastolic, pulse and mean arterial pressures were higher in concentric hypertrophy group compared to the rest.

**Table 3:** Demographic characteristics of different geometric patterns

***Variable***	***Concentric hypertrophy n = 28***	***Eccentric hypertrophy n = 21***	***Concentric remodeling n = 36***	***Normal n = 114***	***P value***
Age (years)	56.07 (6.98)	55.86 (8.01)	56.56 (7.50)	55.97 (7.93)	0.98
Heart Rate (bpm)	83.14 (12.94)	77.05 (12.93)	84.92 (13.27)	80.82 (12.85)	0.12
Weight (kg)	69.21 (10.02)	71.38 (13.06)	69.67 (12.21)	69.25 (10.24)	0.87
Height (m)	1.60 (0.10)	1.61 (0.08)	1.65 (0.07)	1.67 (0.09)	0.002*
BMI (kg/m^2^)	27.29 (4.67)	27.96 (4.27)	26.06 (4.81)	25.09 (3.63)	0.005
SBP (mmHg)	166.07 (18.12)	159.14 (19.59)	148.67 (4.06)	129.75 (22.77)	<0.0001*
DBP (mmHg)	103.21 (8.72)	97.43 (2.54)	91.00 (16.43)	78.00 (14.96)	<0.0001*
Pulse Pressure (mmHg)	62.32 (15.15)	61.71 (14.32)	58.25 (15.50)	51.84 (12.32)	0.0001*
Mean Arterial Pressure (mmHg)	123.99 (10.51)	118.00 (13.16)	110.42 (8.67)	95.28 (16.98)	<0.0001*

bmp = beat per minute, BMI = body mass index, SBP = systolic blood pressure, DBP = diastolic blood pressure.

* = Statistical significance

Table 4 showed that septal and posterior wall thicknesses, end diastolic diameter, left atrial size, LV mass, ejection fraction, stroke volume, cardiac output, RWT, LVM/ht^2.7 ^were significantly different among the various geometric patterns. Septal and posterior wall thicknesses were significantly larger in concentric hypertrophy when compared with other geometric patterns.

**Table 4:** Echo characteristics of different geometric patterns

***Variable***	***Concentric hypertrophy n = 28***	***Eccentric hypertrophy n = 21***	***Concentric remodeling n = 36***	***Normal n = 114***	***P value***
IVSD (cm)	1.24 (0.18)	1.08 (0.18)	1.01 (0.25)	0.84 (0.13)	<0.0001*
PWTD (cm)	1.23 (0.09)	1.01 (0.10)	1.03 (0.15)	0.83 (0.11)	<0.0001*
EDD (cm)	4.49 (0.51)	5.15 (0.27)	3.86 (0.48)	4.63 (0.43)	<0.0001*
LA (cm)	3.45 (0.40)	3.43 (0.43)	3.28 (0.52)	3.16 (0.42)	0.03*
FS (%)	33.43 (5.25)	33.38 (6.50)	35.25 (6.45)	35.70 (6.56)	0.22
EF (%)	67.07 (7.65)	68.95 (9.28)	70.97 (9.04)	72.15 (8.15)	0.03*
RWT	0.56 (0.08)	0.39 (0.04)	0.55 (0.15)	0.36 (0.05)	<0.0001*
LVM (g)	206.60 (39.93)	204.21 (30.50)	126.76 (31.68)	126.90 (27.20)	0.001*
LVM/HT^2.7 ^(g/m^2.7^)	58.19 (8.48)	58.04 (8.95)	33.00 (7.25)	32.26 (6.68)	<0.0001*
SV (ml)	56.29 (16.15)	74.69 (11.71)	40.42 (11.91)	63.88 (16.40)	<0.0001*
CO (L/min)	4.66 (1.47)	5.75 (1.39)	3.42 (1.09)	5.2 (1.70)	<0.0001*

IVSD = Intraventricular septum in diastole, PWTD = Posterior wall thickness in diastole, EDD = End diastolic diameter, LA = Left atrium, FS = Fractional shortening, EF = Ejection fraction, LVM = Left ventricular mass, SV = Stroke Volume, CO = Cardiac Output

* = Statistical significance

## Discussion

Adaptation of the left ventricle to systemic hypertension is complex and it is characterized by functional and structural changes in the left ventricle. The old concept that the heart responds to systemic hypertension by developing concentric or eccentric hypertrophy have been challenged by recent studies[4,6,20]. Hypertension is now known to be associated with a wider spectrum of LV geometric patterns [3,4]. In this study twenty eight percent (28%) of the hypertensive subjects had normal geometry while 72% had various altered geometric while 86% of the controls had normal geometry. Previous studies [20-23] carried out in Brazil, United States and Europe showed different frequencies for various LV geometric patterns. Ethnic differences and environmental factors might play an important role in the different geometric patterns. Also patients with hypertension complicated with coronary artery disease tend to have eccentric hypertrophy predominating[24]. Studies[6,13] involving African American population also depicted the dominance of concentric hypertrophy.

Some authors[23,25] have observed that age significantly affects left ventricular structure and geometric patterns. Sumimoto et al. [26] studied 168 patients with hypertension and were grouped into 3 according to age (young <40, middle-aged and elderly >60). The result showed that the occurrence of normal geometry reduced with age while concentric hypertrophy and concentric remodeling increased with age. However, this study did not reveal any association between age and geometric patterns. The reasons for the lack of relationship are probably due to the narrow age range (56 ± 7 years) in our studied population and the influence of ethnic factor on age-concentricity relation. Studies comparing the LV structure and function among black and white subjects had suggested that RWT and LVM are higher in the blacks [9,11,27]. This observation could have attenuated the age related changes in LV geometric patterns that have been observed in Caucasians.

### Left ventricular structure and function

Left ventricular adaptation to hypertension can present with any of the geometric patterns. The hemodynamic predominance between pressure and volume overload plays an important role in the determination and development of various LV geometric patterns[3]. Concentric remodeling tends to occur early in hypertension due to pressure overload but the left ventricular mass is normal while eccentric hypertrophy is due to volume overload with increased left ventricular mass. Our study showed that in concentric remodeling, chamber dimensions were smaller with reduced stroke volume, cardiac output and increased heart rate compared to the other geometric patterns. Various geometric patterns are also influenced significantly by LV systolic function parameters[28]. Persistent pressure overload in concentric LV geometry with increased total peripheral resistance subsequently impair the systolic function as shown in this study.

### Clinical importance

It is quite significant that seventy-two percent (72%) of our hypertensive subjects had altered geometric patterns. Koren et. al. [4] in a 10 year study in hypertensive patients found that concentric hypertrophy had the highest adverse outcomes (death and morbid events) followed by eccentric and concentric remodeling. Despite the fact that the LV mass is normal in concentric remodeling. It was found to be an important marker of cardiovascular risk. Muiesan et al. [7], also showed that cardiovascular events was more in patients with concentric hypertrophy implicating it as a dangerous adaptative pattern to systemic hypertension. The various geometric patterns are relevant in clinical setting[29] because of their significance and prognostic values [8].

Left ventricular hypertrophy is associated with adverse heart consequences, including reduction in systolic and diastolic function[30], reduced coronary reserve[31], an increased incidence of arrhythmias[32]. Many studies have shown that regression of left ventricular wall thickness and mass occur following antihypertensive therapy [33,34]. This has also been demonstrated after non pharmacological measures to reduce blood pressure e.g. weight reduction and reduction in salt intake[35,36]. Muiesan et al. [7] also demonstrated the favorable prognostic value in geometric patterns following treatment. LV mass reduction improves LV filling and mid-wall fractional shortening, decreases cardiovascular morbidity and mortality and increases coronary reserve. Therefore a targeted therapy at LVH and abnormal LV geometry would be beneficial in management of hypertension.

Since relative wall thickness and left ventricular mass indexation derived from echocardiographic measurements are necessary in determining left ventricular geometric pattern, echocardiographic evaluation of the newly diagnosed hypertensive should be an essential step in their initial workup. In view of the abnormal geometric alterations in newly diagnosed hypertensives noted in this study, longitudinal studies to determine the prognosis of abnormal LV geometry in indigenous Africans are necessary.

## Conclusion

The study, though hospital based (not representing the general population), showed that majority of the newly presenting hypertensives (72%) had abnormal LV geometric patterns. This may argue for the performance of echocardiography in all hypertensive subjects in order to determine the LV geometric pattern. Longitudinal studies of prognosis of abnormal LV geometry are needed to fully evaluate its natural course in indigenous Africans.

## Competing interests

The author(s) declare that they have no competing interests.

## Authors' contributions

AA conceived of the study and participated in the study design and drafted the manuscript, AAA participated in the study design and statistical analysis, OOO took part in the study design and study conception, AD contributed to the study design and statistical analysis, OSO participated in the study design and data acquisition, DBO participated in the study design and data acquisition, AOF conceived of the study and participated in the study design. All authors read and approved the final manuscript.

## Pre-publication history

The pre-publication history for this paper can be accessed here:



## References

[B1] Casale PN, Devereux RB, Milner M, Zullo G, Harshfield GA, Pickering TG, Laragh JH (1986). Value of echocardiographic measurement of left ventricular mass in predicting cardiovascular morbid events in hypertensive men. Ann Intern Med.

[B2] Levy D, Garrison RJ, Savage DD, Kannel WP, Castelli WP (1990). Prognostic implications of echocardiography determined left ventricular mass in the Framingham Heart Study. N Engl J Med.

[B3] Ganau A, Devereux RB, Roman MJ, de Simone G, Pickering TG, Saba PS, Vargiu P, Simongini I, Laragh JH (1992). Patterns of left ventricular hypertrophy and geometric remodeling in essential hypertension. J Am Coll Cardiol.

[B4] Koren MJ, Devereux RB, Casale PN, Savage DD, Laragh JH (1991). Relation of left ventricular mass and geometry to morbidity and mortality in uncomplicated essential hypertension. Ann Intern Med.

[B5] Lin M, Sumimoto T, Hiwada K (1995). Left ventricular geometry and cardiac function in mild to moderate essential hypertension. Hypertens Res.

[B6] Mayet J, Shahi M, Poulter NR, Sever PS, Foale RA, Thom SA (1997). Left ventricular geometry in presenting untreated hypertension. J Hum Hypertens.

[B7] Muiesan ML, Salvetti M, Monteduro C, Bonzi B, Paini A, Viola S, Poisa P, Rizzoni D, Castellano M, Agabiti-Rosei E (2004). Left ventricular concentric geometry during treatment adversely affects cardiovascular prognosis in hypertensive patients. Hypertension.

[B8] Verdecchia P, Schillaci G, Borgioni C, Ciucci A, Battistelli M, Bartoccini C, Santucci A, Santucci C, Reboldi G, Porcellati C (1995). Adverse prognostic significance of concentric remodeling of the left ventricle in hypertensive patients with normal left ventricular mass. J Am Coll Cardiol.

[B9] Mayet J, Shahi M, Foale RA, Poulter NR, Sever PS, Mc GTSA (1994). Racial differences in cardiac structure and function in essential hypertension. Bmj.

[B10] Dunn FG, Oigman W, Sungaard-Riise K, Messerli FH, Ventura H, Reisin E, Frohlich ED (1983). Racial differences in cardiac adaptation to essential hypertension determined by echocardiographic indexes. J Am Coll Cardiol.

[B11] Koren MJ, Mensah GA, Blake J, Laragh JH, Devereux RB (1993). Comparison of left ventricular mass and geometry in black and white patients with essential hypertension. Am J Hypertens.

[B12] Zabalgoitia M, Ur Rahman SN, Haley WE, Oneschuk L, Yunis C, Lucas C, Yarows S, Krause L, Amerena J (1998). Impact of ethnicity on left ventricular mass and relative wall thickness in essential hypertension. Am J Cardiol.

[B13] Fox E, Taylor H, Andrew M, Han H, Mohamed E, Garrison R, Skelton T (2004). Body mass index and blood pressure influences on left ventricular mass and geometry in African Americans: The Atherosclerotic Risk In Communities (ARIC) Study. Hypertension.

[B14] (1992). American Society of Hypertension. Recommendations for routine blood pressure measurement by indirect cuff sphymomanometry. Am J Hypertens.

[B15] Sahn DJ, DeMaria A, Kisslo J, Weyman A (1978). Recommendations regarding quantitation in M-mode echocardiography: results of a survey of echocardiographic measurements. Circulation.

[B16] Devereux RB, Alonso DR, Lutas EM, Gottlieb GJ, Campo E, Sachs I, Reichek N (1986). Echocardiographic assessment of left ventricular hypertrophy: comparison to necropsy findings. Am J Cardiol.

[B17] de Simone G, Daniels SR, Devereux RB, Meyer RA, Roman MJ, de Divitiis O, Alderman MH (1992). Left ventricular mass and body size in normotensive children and adults: assessment of allometric relations and impact of overweight. J Am Coll Cardiol.

[B18] Savage DD, Garrison RJ, Kannel WB, Levy D, Anderson SJ, Stokes J, Feinleib M, Castelli WP (1987). The spectrum of left ventricular hypertrophy in a general population sample: the Framingham Study. Circulation.

[B19] Teichholz LE, Kreulen T, Herman MV, Gorlin R (1976). Problems in echocardiographic volume determinations: echocardiographic-angiographic correlations in the presence of absence of asynergy. Am J Cardiol.

[B20] Cunha DM, da Cunha AB, Martins WA, Pinheiro LA, Romeo LJ, de Moraes AV, Morcerf FP (2001). Echocardiographic assessment of the different left ventricular geometric patterns in hypertensive patients. Arq Bras Cardiol.

[B21] Roman MJ, Pickering TG, Schwartz JE, Pini R, Devereux RB (1996). Relation of arterial structure and function to left ventricular geometric patterns in hypertensive adults. J Am Coll Cardiol.

[B22] Bugra Z, Koylan N, Vural A, Erzengin F, Umman B, Yilmaz E, Meric M, Buyukozturk K (1998). Left ventricular geometric patterns and QT dispersion in untreated essential hypertension. Am J Hypertens.

[B23] Shipilova T, Pshenichnikov I, Kaik J, Volozh O, Abina J, Kalev M, Lass J, Meigas K (2003). Echocardiographic assessment of the different left ventricular geometric patterns in middle-aged men and women in Tallinn. Blood Press.

[B24] Zabalgoitia M, Berning J, Koren MJ, Stoylen A, Nieminen MS, Dahlof B, Devereux RB (2001). Impact of coronary artery disease on left ventricular systolic function and geometry in hypertensive patients with left ventricular hypertrophy (the LIFE study). Am J Cardiol.

[B25] de Simone G, Daniels SR, Kimball TR, Roman MJ, Romano C, Chinali M, Galderisi M, Devereux RB (2005). Evaluation of concentric left ventricular geometry in humans: evidence for age-related systematic underestimation. Hypertension.

[B26] Sumimoto T, Mukai M, Murakami E, Kokubu T, Lin M, Shigematsu Y, Hamada M, Hiwada K (1995). Effect of age on left ventricular geometric patterns in hypertensive patients. J Hypertens.

[B27] Hinderliter AL, Blumenthal JA, Waugh R, Chilukuri M, Sherwood A (2004). Ethnic differences in left ventricular structure: relations to hemodynamics and diurnal blood pressure variation. American Journal of Hypertension.

[B28] de Simone G, Devereux RB, Celentano A, Roman MJ (1999). Left ventricular chamber and wall mechanics in the presence of concentric geometry. J Hypertens.

[B29] de Simone G (2004). Concentric or eccentric hypertrophy: how clinically relevant is the difference?. Hypertension.

[B30] Slama M, Susic D, Varagic J, Frohlich ED (2002). Diastolic dysfunction in hypertension. Curr Opin Cardiol.

[B31] Pichard AD, Gorlin R, Smith H, Ambrose J, Meller J (1981). Coronary flow studies in patients with left ventricular hypertrophy of the hypertensive type. Evidence for an impaired coronary vascular reserve. Am J Cardiol.

[B32] Messerli FH, Ventura HO, Elizardi DJ, Dunn FG, Frohlich ED (1984). Hypertension and sudden death. Increased ventricular ectopic activity in left ventricular hypertrophy. Am J Med.

[B33] Dahlof B, Pennert K, Hansson L (1992). Reversal of left ventricular hypertrophy in hypertensive patients. A metaanalysis of 109 treatment studies. Am J Hypertens.

[B34] Schmieder RE, Martus P, Klingbeil A (1996). Reversal of left ventricular hypertrophy in essential hypertension. A meta-analysis of randomized double-blind studies. Jama.

[B35] MacMahon SW, Wilcken DE, Macdonald GJ (1986). The effect of weight reduction on left ventricular mass. A randomized controlled trial in young, overweight hypertensive patients. N Engl J Med.

[B36] Ferrara LA, de Simone G, Pasanisi F, Mancini M (1984). Left ventricular mass reduction during salt depletion in arterial hypertension. Hypertension.

